# Symbiotic microbiome *Staphylococcus aureus* from human nasal mucus modulates IL-33-mediated type 2 immune responses in allergic nasal mucosa

**DOI:** 10.1186/s12866-020-01974-6

**Published:** 2020-10-07

**Authors:** Yung Jin Jeon, Chan Hee Gil, Jina Won, Ara Jo, Hyun Jik Kim

**Affiliations:** 1 Department of Otorhinolaryngology, Gyeongsang National University Hospital, Jinju, Republic of Korea; 2 Department of Otorhinolaryngology - Head and Neck Surgery, Seoul National University College of Medicine, 103, Daehak-ro, Jongno-gu, Seoul, 03080 Republic of Korea; 3 Department of Otorhinolaryngology, Seoul National University Hospital, Seoul, Republic of Korea

**Keywords:** *Staphylococcus aureus*, Allergic rhinitis, Interleukin-33, Symbiosis, Nasal microbiome

## Abstract

**Background:**

The host-microbial commensalism can shape the innate immune responses in respiratory mucosa and nasal microbiome also modulates front-line immune mechanism in the nasal mucosa. Inhaled allergens encounter the host immune system first in the nasal mucosa, and microbial characteristics of nasal mucus directly impact the mechanisms of initial allergic responses in nasal epithelium. However, the roles of the nasal microbiome in allergic nasal mucosa remain uncertain. We sought to determine the distribution of nasal microbiomes in allergic nasal mucosa and elucidate the interplay between nasal microbiome *Staphylococcus* species and Th2 cytokines in allergic rhinitis (AR) models.

**Results:**

*Staphylococcus aureus* (AR-SA) and *S. epidermidis* (AR-SE) were isolated from the nasal mucosa of patients with AR. The influence of nasal microbiome *Staphylococcus* species on allergic nasal mucosa was also tested with in vitro and in vivo AR models. Pyrosequencing data showed that colonization by *S. epidermidis* and *S. aureus* was more dominant in nasal mucus of AR subjects. The mRNA and protein levels of IL-33 and TSLP were significantly higher in AR nasal epithelial (ARNE) cells which were cultured from nasal mucosa of AR subjects, and exposure of ARNE cells to AR-SA reduced IL-33 mRNA and secreted protein levels. Particularly, ovalbumin-driven AR mice inoculated with AR-SA by intranasal delivery exhibited significantly reduced IL-33 in their nasal mucosa. In the context of these results, allergic symptoms and Th2 cytokine levels were significantly downregulated after intranasal inoculation of AR-SA in vivo AR mice.

**Conclusion:**

Colonization by *Staphylococcus* species was more dominant in allergic nasal mucosa, and nasal commensal *S. aureus* from subjects with AR mediates anti-allergic effects by modulating IL-33-dependent Th2 inflammation. The results demonstrate the role of host-bacterial commensalism in shaping human allergic inflammation.

## Background

Allergic rhinitis (AR), an IgE- and T helper (Th)2-mediated inflammatory nasal disease, is caused by sensitized immune responses to inhaled allergens. This allergen-specific immune response is thought to arise from an imbalance in Th1-Th2 immune regulation that results in increased levels of Th2 cytokines [1, 2]. Nasal epithelial cells exposed to external allergens induce Th2 inflammatory responses and Th2 inflammations proceed to the upper airway mucosa. The allergen-mediated inflammatory immune response begins with increased secretion of epithelial cell-derived cytokines, such as thymic stromal lymphopoietin (TSLP) and interleukin (IL)-33 [1]. Epithelial cell-derived cytokines provide critical signals to innate and adaptive cell populations related to Th2 inflammation [2, 3]. Allergic nasal epithelium might be responsible for the vast majority of allergic inflammation to inhaled allergens, and research about regulation of epithelial cell-derived cytokines is needed to develop a more effective approach to treatment of AR [4]. IL-33, which is produced by the airway epithelium and other cell types, is a key cytokine involved in allergic airway diseases and provides an essential axis for rapid immune responses and tissue homeostasis. Since the discovery of IL-33, tremendous progress has been made in understanding its biological functions, and the potential importance of IL-33 as a therapeutic target of AR has been suggested [5, 6].

Human mucosal surfaces are in direct contact with the external environment and are susceptible to invasion and colonization by various allergens and pathogens [7–9]. Respiratory mucosa is constantly exposed to inhaled pathogens and allergens that directly impact mucosal immune mechanisms [10, 11]. Studies on interaction between the mucosal microbiome and the host increasingly consider the contribution of mucosal immune responses and specific microbiome-mediated protection from external pathogens to integrate environmental signals [11]. Inhaled allergens encounter the host immune system first in the nasal mucosa, and microbial characteristics of nasal mucus directly impact the mechanisms of initial allergic responses in nasal epithelium [9, 12, 13]. Insights into the microbiota and dysbiosis of allergic nasal mucosa provide fundamental information regarding susceptibility to allergens and their relation to allergic inflammation, including induction of epithelial cell-derived Th2 cytokines such as IL-33 [14–16]. There is considerable evidence that the microbiota contributes to responses of the mucosal immune system and protects against pathogenic bacterial or viral infection. Recent studies have sought to identify how the commensal microbiota regulates sensitization tolerance to allergens in asthma [9, 17, 18]. However, our knowledge of microbial composition in allergic nasal mucus is limited, and the responses of the nasal microbiome to inhaled allergens have not been comprehensively examined in nasal mucosa.

Based on our previous evidence of microbial distribution in healthy nasal mucus, we assessed the microbial composition in allergic nasal mucus and subsequently investigated whether nasal commensal bacteria in allergic nasal mucus contributes to Th2 inflammatory responses in nasal epithelium. The present study identified *Staphylococcus* species, including *S. epidermidis* and *S. aureus*, as the most abundant constituents in allergic nasal mucus. Particularly, *S. aureus* isolated from the nasal mucus of AR subjects accelerated the reduction of IL-33 in AR nasal epithelium. Furthermore, allergic nasal commensal *S. aureus* prevented allergic inflammation in vivo in mice by reducing IL-33-related Th2 immune responses in the nasal mucosa. Overall, our study presents evidence that *Staphylococcus* species colonization is significantly increased in the nasal mucus of AR subjects. In addition, administration of *S. aureus* isolated from human allergic nasal mucus suppressed allergic inflammation in nasal mucosa by reducing IL-33 secretion.

## Materials and methods

Additional methodological details are available in the online supplement.

### Participant recruitment

Information on 17 subjects with AR enrolled in this study and the exclusion criteria are described in the online data supplement. Participation was voluntary, and written informed consent was obtained from all subjects. The Institutional Review Board of Seoul National University College of Medicine (No. 1709–049-883) and Gyeongsang National University Hospital (No. 2019–05-004) approved the protocol for this study.

### Sample collection

Mucus and/or nasal mucosa from the middle turbinate of human subjects was collected and assessed for quality as described in the online data supplement (supplementary video file).

### The characterization of staphylococcus species in human allergic nasal mucosa

To isolate bacterial colonies, the mucus was placed in lysogeny broth (LB) plates. After 1 day of incubation, bacterial colonies were obtained from LB plates. The species of each colony was identified using GS-FLX 454 pyrosequencing with 16S rRNA gene amplification, as described previously [19]. *Staphylococcus epidermidis* and *S. aureus* strains from five individual subjects with AR were used in the study.

### Cell culture

Normal human nasal epithelial (NHNE) and allergic rhinitis nasal epithelial (ARNE) cells were each cultured from five subjects using an air-liquid interface method. Cells were used 14 days after creation of the air-liquid interface (ALI). Isolated AR-SA and AR-SE were inoculated as described previously [19]. Details are available in the online supplement.

### Murine inoculation model

Experiments with four-week-old female wild type (WT) BALB/c mice (Orient, Gyeonggi, Republic of Korea) were carried out according to guidelines approved by the Institutional Animal Care and Use Committees of Seoul National University Hospital (No. 2016–1470). Microbiome depletion, AR-SA and AR-SE inoculation, nasal lavage (NAL) sample collection, and nasal tissue harvesting are described in the online supplement.

Mice were divided into four groups depending on *S. aureus* inoculation. The negative control group (WT) was sensitized and challenged with phosphate-buffered saline (PBS), and the positive control group (AR-OVA) was sensitized and challenged with ovalbumin (OVA). Both WT mice and AR-OVA mice were inoculated with human nasal microbiome *S. aureus* by intranasal delivery. The schematic figure for allergen sensitization, intranasal challenge, and *S. aureus* inoculation is shown in Fig. 4a. Mice were euthanized and sacrificed by cervical dislocation and by intramuscular injection of high dose of a mixture of 10 mg/kg xylazine (Bayer, Puteaux, France) and 5 mg/kg ketamine (Merial, Lyon, France) according to the reviewed protocol. Death was verified when no heartbeat was detected. There were no mice without euthanasia. When mice were euthanized by injection, a cervical dislocation was also performed to ensure that the mice were dead.

### Serum levels of total and OVA-specific IgE

AR mouse serum samples were stored at − 70 °C before use for measurements of total IgE and OVA specific IgE, as described previously [20]. Details are available in the online supplement.

### Histologic analysis

Mice heads were fixed in 10% formalin, decalcified, and embedded in paraffin wax. Nasal tissues were sectioned and stained with hematoxylin and eosin (H&E) for inflammatory cell counting, Sirius red stain for eosinophil counting, and periodic acid-Schiff (PAS) stain to indicate secretory cells of the nasal epithelium.

### Colony count

Bacterial samples were serially diluted ten-fold with phosphate-buffered saline (PBS). Next, 10 μl of each diluted sample was plated on an LB agar plate. The plates were incubated for 24 h at 37 °C. The numbers of AR-SA and AR-SE colonies growing on the agar surface were counted manually. Bacterial growth was reported based on the colony-forming units (CFUs) for each sample.

### Real-time PCR and RNA preparation

Levels of transcripts encoding human IL-33 and TSLP, mouse IL-4, IL-5, IL-13, and IL-33, or *femA*s specific for *S. aureus* (*femA-SA*) and *S. epidermidis* (*femA-SE*) were determined using real-time PCR (RT-PCR), as described in the online supplement.

### Protein isolation and Western blot

IL-33 levels were assessed by western blotting using human IL-33 antibody (AF3625, R&D Systems, Minneapolis, MN, USA). Details are available in the online supplement.

### Quantification of secreted cytokines

Secreted mouse IL-33 (DY3626) was quantified using the DuoSet® ELISA kit from R&D Systems according to the manufacturer’s instructions. To quantify IL-33, nasal lavage (NAL) fluid of a murine model was collected. The working range of the assay was 15.6–1000 pg/mL.

### Statistical analyses

At least three independent experiments were performed with cultured cells from each donor, and data are expressed as mean ± standard deviation (SD) of three independent experiments. Groups were compared by the Mann-Whitney U test, and differences among treatment groups were evaluated by analysis of variance (ANOVA) with a post hoc test. A *P*-value of < 0.05 was considered statistically significant. All statistical analyses were performed with GraphPad Prism software (version 5; GraphPad Software, Inc., La Jolla, CA, USA).

## Results

### Characterization of bacterial communities in allergic nasal mucus

**Fig. 1:**
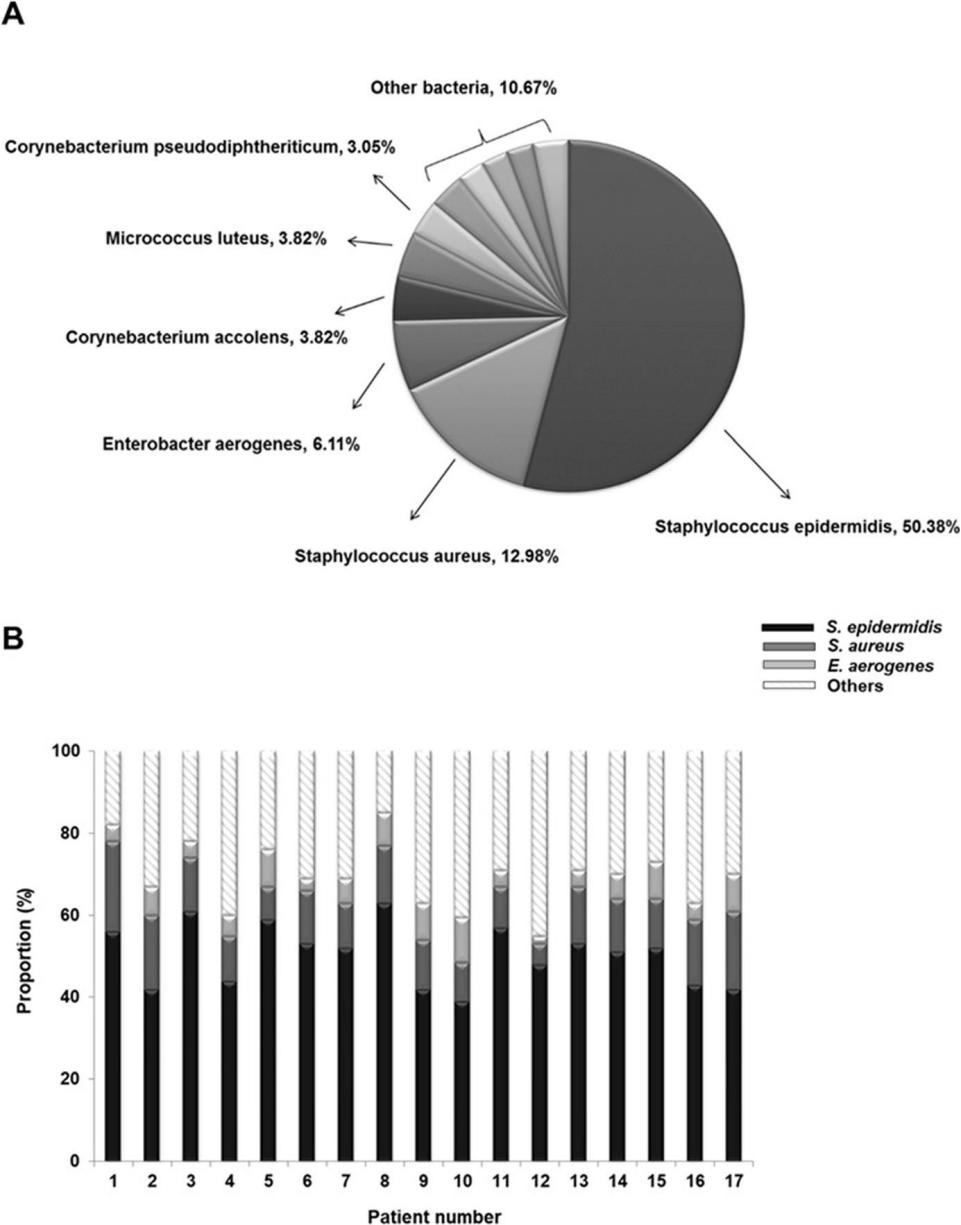
Distribution of bacterial colonies in nasal mucus of subjects with allergic rhinitis (AR). **a** Bacterial species cultured from mucus samples obtained from middle turbinates of subjects with allergic rhinitis (AR) (*N* = 17) were identified via 16S rRNA gene sequencing. Distribution of the 112 identified bacterial species is presented in the graph. **b** The relative abundance of nasal commensal, such as *S. epidermidis, S. aureus, E. aerogenes* and others, in each AR subjects. The bar graph presents the relative abundance of nasal commensal organisms of 17 allergic rhinitis subjects at the species level

The microbial compositions of middle turbinate mucus in human subjects with AR (*N* = 17) were evaluated by analyzing cultured bacterial colonies and 16S rRNA gene sequencing, as previously described [6]. Based on at least 97% sequence identity, 112 bacterial species were discovered in middle turbinate mucus of subjects with AR. *Staphylococcus epidermidis, S. aureus*, *Enterobacter aerogenes, Corynebacterium accolens, Micrococcus luteus, Corynebacterium pseudodiphtheriticum,* and *Klebsiella pneumoniae* were among the most commonly detected species. *S. epidermidis* demonstrated the highest abundance, accounting for 58.04% of mapped sequences, and *S. aureus* exhibited the largest increase in presence of microbial distribution in allergic nasal mucus (14.29% in AR vs 5.61% in healthy nasal mucus [6]) (Fig. 1a). The proportion of *S. epidermidis* and *S aureus* in allergic nasal mucus varied widely among subjects with AR to a maximum of 79.1%. The lowest abundance of *Staphylococcus* species in the nasal mucus of subjects with AR was 48.9% (Fig. 1b). We then questioned the role of the nasal commensal organisms *S. epidermidis* (AR-SE) and *S. aureus* (AR-SA) in allergic nasal mucus. Both AR-SE and AR-SA strains were isolated from four AR subjects to assess the mechanistic link between dominant allergic nasal commensals and pathogenesis of AR in nasal mucosa.

### Inoculation of nasal commensal *S. aureus* suppressed IL-33 secretion in ARNE cells

**Fig. 2:**
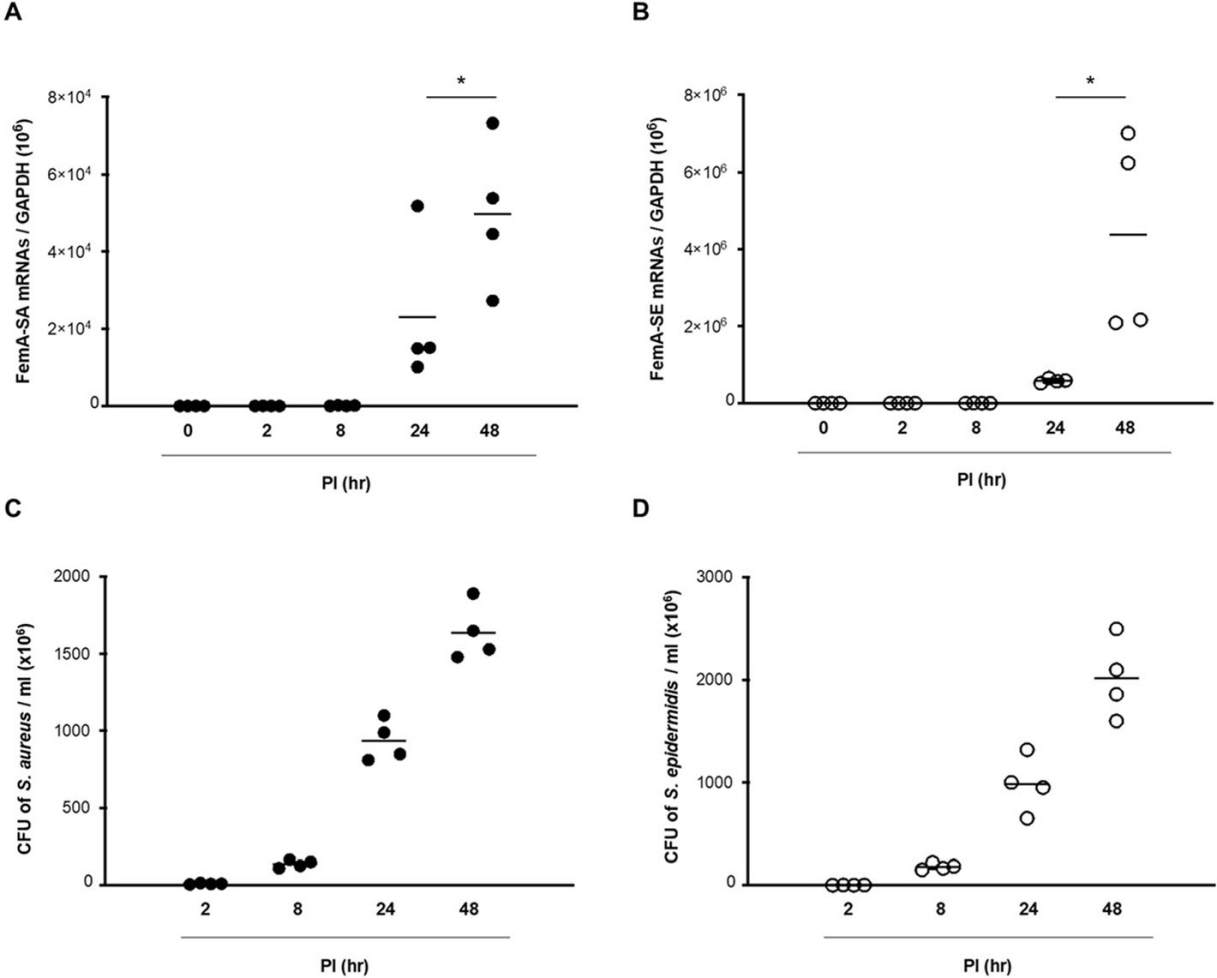
Susceptibility of nasal commensal *Staphylococcus* species to allergic rhinitis nasal epithelial (ARNE) cells. ARNE cells from four AR subjects were inoculated with nasal commensal, *S. aureus* and *S. epidermidis* at a multiplicity of infection (MOI) of 0.25. The mRNA levels of *femA* genes specific for *S. aureus* (**a**) and *S. epidermidis* (**b**), normalized to cellular GAPDH transcript levels, were monitored by real-time PCR for 48 h post-infection. Colony counts of supernatants in cultured ARNE cells inoculated with nasal commensal *S. aureus* (**c**) and *S. epidermidis* (**d**) from allergic rhinitis subjects were performed. Results are presented as mean ± standard deviation (SD) from five independent experiments. *p < 0.05 compared with control ARNE cells

**Fig. 3:**
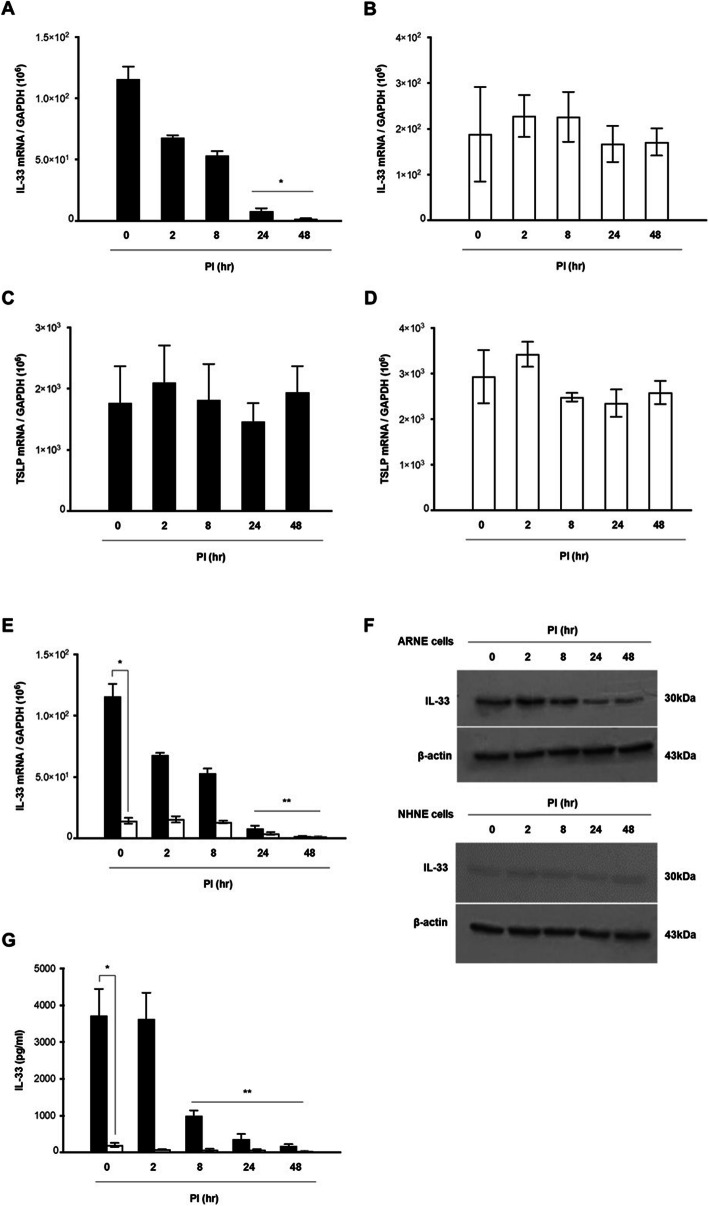
*Staphylococcus* species suppress expression of interleukin-33 (IL-33). ARNE cells were inoculated with nasal commensal *S. aureus* (AR-SA) and *S. epidermidis* (AR-SE) from subjects with AR at a MOI of 0.25. Levels of mRNAs encoding epithelial cell-derived Th2 cytokines including IL-33 and thymic stromal lymphopoietin (TSLP) were monitored by real-time PCR (IL-33 mRNA in AR-SA-inoculated ARNE cells (**a**) and AR-SE-inoculated ARNE cells (**b**), TSLP mRNA in AR-SA-inoculated ARNE cells (**c**) and AR-SE-inoculated ARNE cells (**d**). IL-33 mRNA and protein levels of normal human nasal epithelial (NHNE, white bar) and ARNE cells (Black bar) inoculated with AR-SA were analyzed by real-time PCR (e) and Western blot (**f**), respectively. Secreted IL-33 protein levels of normal human nasal epithelial (NHNE, white bar) and ARNE cells (Black bar) inoculated with AR-SA were analyzed by ELISA (**g**) Results are presented as mean ± standard deviation (SD) from five independent experiments and western blot results is also representative of five independent experiments. *p < 0.05 compared with control ARNE cells (a, b, c, and d). *p < 0.05 comparing levels between NHNE and ARNE cells (e, g). The original, unprocessed versions of full-length gel/blot images are included in the online data supplement (Additional file 3: supplementary figure)

The kinetics of expression of epithelial cell-derived Th2 cytokines genes, protein production, and secreted protein levels were studied in ARNE cells after inoculation with dominant nasal microbiome AR-SE and AR-SA in human allergic nasal mucosa. Cultured ARNE cells from five subjects with AR were inoculated with AR-SA and AR-SE at an MOI of 0.25, and cell lysate and supernatant were harvested at 0, 2, 8, 24, and 48 h post of inoculation (hpi). The mRNA levels of *femA-SA* and *femA-SE* genes were then measured using RT-PCR. The results showed that *femA-SA* and *femA-SE* mRNA levels increased significantly from 8 h after inoculation, with the highest expression observed at 48 hpi (Fig. 2a, b). In addition, AR-SA colony counts from the supernatant of ARNE were significantly increased at 8 hpi (1.05 × 10^8^ ± 7.07 × 10^6^ CFU/mL) and were the highest at 48 hpi (1.28 × 10^9^ ± 3.20 × 10^8^ CFU/mL) (Fig. 2c). AR-SE colony counts from supernatant of ARNE cells were also significantly increased at 8 hpi (1.35 × 10^8^ ± 2.12 × 10^7^ CFU/mL) and were the highest at 48 hpi (1.85 × 10^9^ ± 3.53 × 10^8^ CFU/mL) (Fig. 2d). Then, mRNA levels of IL-33 and TSLP were measured to analyze the effects of nasal commensal *Staphylococcus* species inoculation on the expression of epithelial cell-derived Th2 cytokines in ARNE cells. Although IL-33 mRNA levels were also slightly attenuated in AR-SE inoculated ARNE cells at 48 hpi, IL-33 mRNA levels of ARNE cells decreased more significantly after inoculation of AR-SA from 8 hpi, and the lowest level was observed in AR-SA-inoculated ARNE cells at 48 hpi (Fig. 3a, b). TSLP mRNA levels did not significantly change in ARNE cells inoculated with AR-SA or AR-SE (Fig. 3c, d).

We then tested whether *S. aureus* targets IL-33 gene and protein expression in ARNE cells and compared expression levels in ARNE cells with those of AR-SA-inoculated NHNE cells. The IL-33 mRNA levels of ARNE cells were significantly higher than those of NHNE cells regardless of AR-SA inoculation (mean value of ARNE 1.21 × 10^8^ vs mean value of NHNE 3.82 × 10^6^). Reduced *IL-33* gene expression was only observed in AR-SA inoculated ARNE cells until 48 h after inoculation (mean value: 3.21 × 10^3^, Fig. 3e). Western blot analysis similarly revealed that full-length IL-33 protein levels gradually decreased until 48 h in AR-SA-inoculated ARNE cells, and AR-SA did not show any IL-33 reduction in NHNE cells (Fig. 3f). The secreted protein levels of IL-33 in the supernatant of ARNE cells was measured using ELISA and compared with those of NHNE cells after AR-SA inoculation. The secreted protein concentration of IL-33 was significantly higher in the supernatant of ARNE cells (mean value of ARNE: 3693.2 pg/ml vs mean value of NHNE: 231.4 pg/ml) and secreted protein concentration of IL-33 was reduced in the supernatant of AR-SA-inoculated ARNE cells until 48 h after inoculation (mean value: 103.5 pg/ml, Fig. 3g). Based on these findings, nasal microbiome *S. aureus* isolated from the nasal mucus of subjects with AR reduced epithelial cell-derived Th2 cytokines, IL-33 gene expression, and protein production in allergic nasal epithelium.

### Pretreatment with human nasal microbiome *S. aureus* suppresses allergic inflammation in vivo

**Fig. 4:**
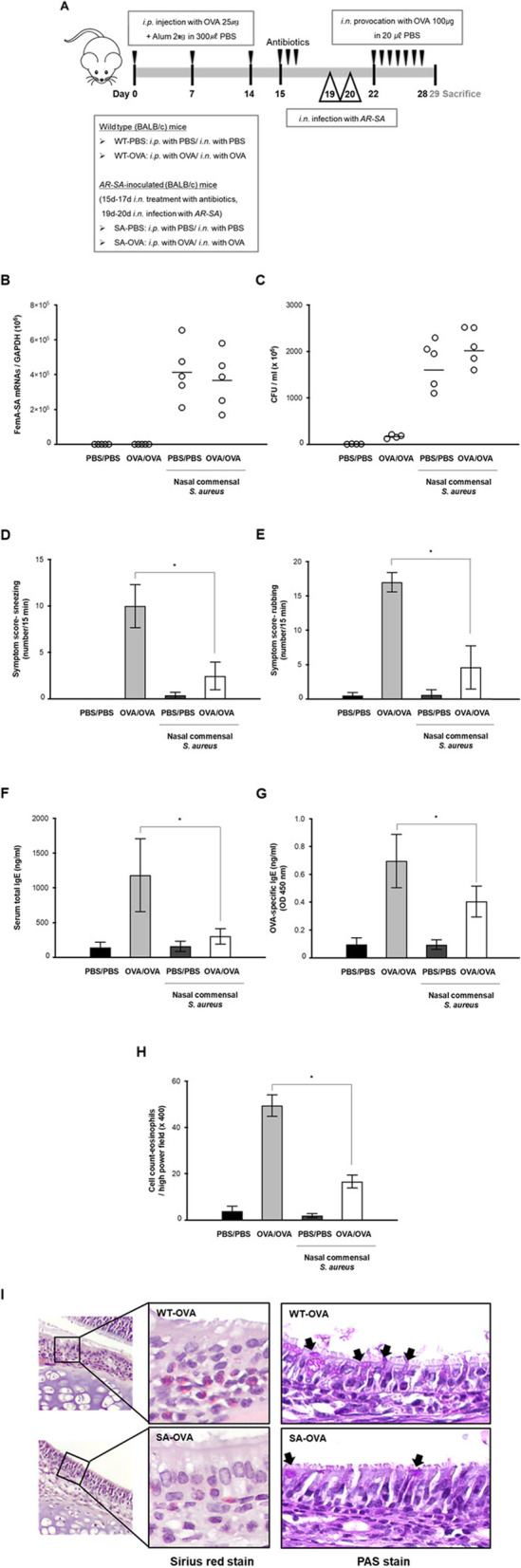
Experimental protocol and comparison of nasal symptoms, serum immunoglobulin E (IgE) levels, and eosinophilic infiltration levels. **a** The experimental protocol for development of allergic asthmatic mouse using BALB/C. Mice were sensitized by intraperitoneal injection of ovalbumin mixed with aluminum hydroxide on days 0, 7, and 14. Daily OVA intranasal challenge was performed from days 22 to 28 (OVA/OVA). Human nasal *S. aureus* (AR-SA) (3.2 × 10^6^ CFU/30 μl. PBS) was inoculated at indicated time points (day 19, 20). **b** mRNA levels of femA gene, specific for *S. aureus* and normalized to cellular GAPDH transcript levels, were monitored by real-time PCR. **c** Colony counts of nasal lavage fluid were performed. Frequencies of sneezing (**d**) and rubbing (**e**) events were assessed over a 15 min period after OVA provocation. Serum levels of total IgE (**f**) and OVA-specific IgE (**g**) were significantly lower in SA-OVA mice than in WT-OVA mice (*N* = 5). Histologic findings in nasal mucosa of each group (× 400 magnification) with Sirius red staining for eosinophils (**h**) and periodic acid-Schiff (PAS) staining for secretory cells (**i**). Results are presented as mean ± standard deviation (SD) (*N* = 5) and histologic finding is representative of nose sections from five mice. *p < 0.05

Considering the in vitro effect of nasal commensal *S. aureus* on IL-33 expression in ARNE cells, we next assessed the anti-allergic effect of *S. aureus* using an in vivo murine model of AR. BALB/C mice were sensitized and challenged with OVA (AR-OVA, *N* = 5). BALB/C mice (N = 5) were inoculated with human nasal microbiome *S. aureus* at 2 days (day 19, 20) following nasal microbiota depletion (day 15, 16, 17) using 30 μl of an antibiotic cocktail (vancomycin, neomycin, ampicillin, and metronidazole) (Fig. 4a). Nasal symptoms such as sneezing and rubbing, total serum IgE, OVA-specific IgE, serum eosinophil count, and histologic findings of nasal mucosa were compared for WT and AR-OVA mice depending on human nasal AR-SA.

First, *S. aureus* mRNA levels were measured in in vivo nasal mucosa to confirm effective colonization of AR-SA in WT mice and AR-OVA mice after inoculation. RT-PCR results revealed that *femA* mRNA levels and colony counts of *S. aureus* were significantly higher in WT and AR-OVA mice with *S. aureus* inoculation than in WT and AR-OVA groups without inoculation (Fig. 4b, c). The difference in allergic symptoms of in vivo AR mice was clearly observed depending on inoculation with *S. aureus*-inoculated AR-OVA mice. The frequencies of sneezing and nasal rubbing in the positive control group were 9.98 ± 2.31 (WT mice) and 17.01 ± 1.43 (AR-OVA), respectively. These scores were significantly reduced in AR-OVA mice with human nasal microbiome AR-SA inoculation (1.35 ± 0.61, *p* = 0.018, and 4.60 ± 3.14, *p* = 0.010, respectively) (Fig. 4d, e). Serum levels of total IgE (Fig. 4f) and OVA-specific IgE (Fig. 4g) were significantly lower in AR-OVA mice compared with the WT group. Eosinophils and secretory cells of nasal mucosal tissue were detected by Sirius red and PAS staining. Eosinophil infiltration in the submucosa of AR-OVA mice was significantly higher than that in *S. aureus*-inoculated AR-OVA mice (Fig. 4h). Fewer PAS-stained secretory cells were observed in nasal mucosal tissue of *S. aureus*-inoculated AR-OVA mice than in AR-OVA mice (Fig. 4i). Collectively, these data suggest that intranasal inoculation of human microbiome *S. aureus* improved allergic inflammation in an in vivo AR model. In addition, nasal microbiome isolated from allergic nasal mucus reduced Th2 inflammation of in vivo nasal mucosa.

**Fig. 5:**
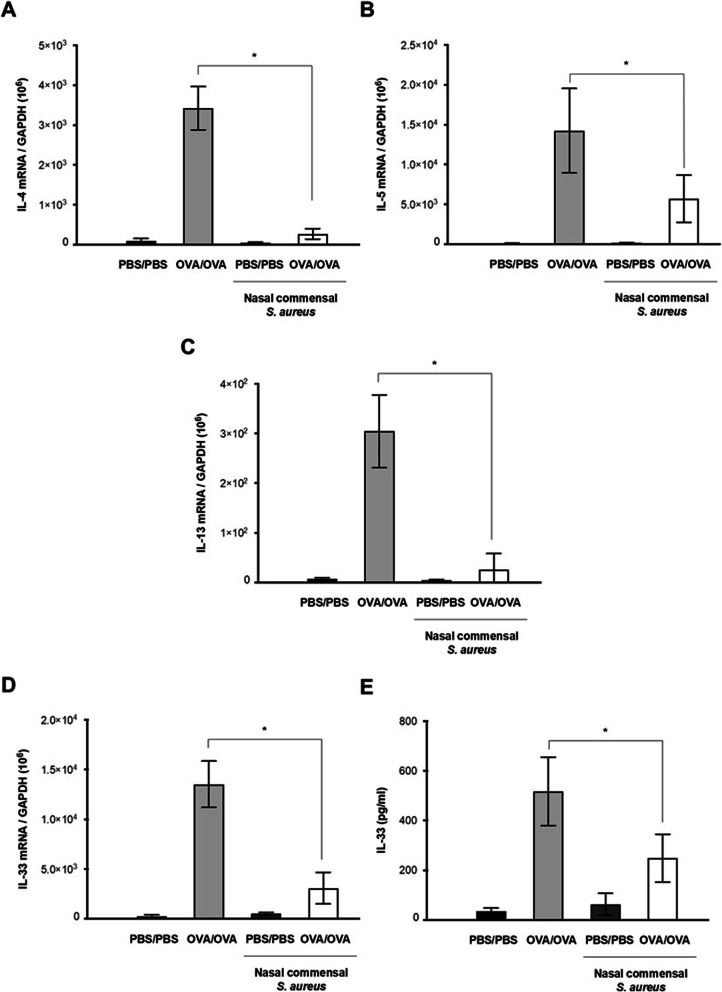
Comparison of cytokine mRNA levels in nasal mucosa. Wild type mice (PBS/PBS) and AR mice (OVA/OVA) were inoculated with human nasal *S. aureus* (3.2 × 10^6^ CFU/30 μl PBS) at indicated time points (day 19, 20) and mRNA levels of interleukin (IL)-4 (**a**), IL-5 (**b**), IL-13 (**c**), and IL-33 (**d**) were measured by real-time PCR. **e** Secreted IL-33 protein levels secreted from nasal mucosa were measured by ELISA using nasal lavage fluid. Results are presented as mean ± standard deviation (SD) (*N* = 5). *p < 0.05

Finally, mRNA expression of IL-4, IL-5, and IL-13 was measured to evaluate changes in Th2 cytokine-regulated inflammation of in vivo nasal mucosa after *S. aureus* inoculation. As expected, intranasal inoculation of AR-SA significantly reduced the mRNA levels of IL-4, IL-5, and IL-13 in in vivo nasal mucosa of AR-OVA mice compared to AR-OVA mice without AR-SA inoculation (Fig. 5a, b and c). Levels of IL-33 mRNA in mouse nasal mucosa were then determined by RT-PCR. Secreted IL-33 protein levels in NAL fluid were also determined with ELISA in in vivo AR mice depending on human nasal *S. aureus* inoculation. RT-PCR results revealed a higher level of IL-33 mRNA in the nasal mucosa of AR-OVA mice that was significantly attenuated in the nasal mucosa of AR-OVA mice with AR-SA inoculation (Fig. 5d). In addition, OVA-induced secreted IL-33 protein level was significantly decreased in WT-OVA mice with inoculation of human nasal microbiome *S. aureus* (Fig. 5e). These findings demonstrate that the nasal mucosa of AR mice had significantly reduced Th2 cytokines in response to human nasal microbiome *S. aureus. S. aureus*-regulated anti-allergic immune responses might be involved in IL-33 downregulation of the nasal mucosa in the in vivo AR model.

## Discussion

Here, we showed that there was significantly more colonization by *S. epidermidis* and *S. aureus* in the nasal mucus of subjects with AR compared to healthy nasal mucus. In addition, inoculation of human nasal microbiome *S. aureus* to ARNE cells and an OVA-sensitized AR murine model reduced Th2 cytokine-regulated allergic inflammation by suppressing the epithelial cell-derived cytokine IL-33. Our findings imply that intranasal administration of *S. aureus* is a potential strategy for improving allergic inflammation by regulating epithelial cell-derived cytokines. Inoculation of *S. aureus* isolated from nasal mucus of subjects with AR might be a new treatment for controlling allergic inflammation caused by inhaled allergens in the nasal epithelium.

Respiratory epithelium is the first target organ for environmental allergens, and recent work has highlighted the critical role of the respiratory epithelium as a barrier that restricts host exposure of allergens [11, 21–23]. Like intestinal symbiotic commensal organisms, the microbiome that colonizes respiratory mucus is thought to have critical immune functions in the respiratory tract. There may be a functional connection between respiratory epithelial cells that contact inhaled allergens and the respiratory microbiome. There is increasing interest in compositional and predicted functional differences in respiratory mucus microbiomes of allergic diseases. The importance of the respiratory microbiome, especially with respect to Th2 cytokine-regulated immune responses, has been increasingly recognized [24–28]. Understanding compositional changes in the microbiome and identifying dominant microbial species in respiratory mucus might be essential for developing new therapeutic approaches for allergic respiratory diseases. The nasal mucosa is also a key player in immunologic defenses that protect the respiratory tract and is responsible for filtering inhaled allergens from direct exposure to pressurized airflow [22, 23].

Diverse evidence suggests that microbes reside in the nasal mucus secreted from epithelial cells. Our previous study revealed that the most dominant nasal microbiome, *S. epidermidis*, increased immunity against influenza viral lung infection through IFN-λ amplification at the level of the nasal epithelium. The findings provide evidence that the nasal microbiome strengthens innate immune responses of the host to protect the respiratory tract from viral pathogens that it is exposed to [17]. We extended this paradigm to the nasal microbiome in the field of allergic diseases and noted marked changes in the microbial composition of nasal mucus in subjects with AR. The distribution of both *S. epidermidis* and *S. aureus* was significantly increased in AR nasal mucus to constitute over 60% of the identified nasal microbiome. Among nasal microbiomes of AR nasal mucus, *S. aureus* colonization increased the most compared to normal mucus. Thus, we focused on the contributions of *S. epidermidis* and *S. aureus* to the pathogenic mechanism of AR and synergism of *Staphylococcus* species colonization with Th2-related allergic reaction in the nasal mucosa. Both *S. epidermidis* and *S. aureus* are well-known human pathogens that may cause serious opportunistic infections of the respiratory tract. However, *Staphylococcus species* are commonly detected in the normal microflora of the skin, intestine, and upper airway and many studies implicate the role of *Staphylococcus* species as symbiotic commensal organisms that are also involved in allergic diseases [29–31]. Patients with allergic eczema are more likely to have skin colonized with *S. aureus* than healthy control subjects, and disease severity is associated with *S. aureus* colonization. The dominant distribution of *S. aureus* in the upper respiratory tract of patients with asthma and aspirin sensitivity has been reported up to 87.5%, and higher nasal *S. aureus* colonization is significantly related to asthma [29, 32]. In addition, patients with AR are more frequently colonized with nasal *S. aureus* or sensitized to *S. aureus* enterotoxins than healthy control subjects [30]. Therefore, we hypothesized that *Staphylococcus* species aggravate allergic immune responses in nasal epithelium, inoculation of *Staphylococcus* colonies dysregulates Th2 inflammation, and epithelial cell-derived Th2 cytokines of in vitro and in vivo AR models would be elevated. We investigated the definite function of *Staphylococcus* species in allergic nasal mucus by isolating colonies of *S. epidermidis* and *S. aureus* from human nasal mucus of subjects with AR.

Interestingly, the results differed from what was initially expected. Nasal commensal *S. aureus* isolated from the nasal mucus of subjects with AR reduced IL-33 expression in both ARNE cells and the OVA-driven AR mice model. Conversely, *S. epidermidis* isolated from the nasal mucus of subjects with AR did not induce significant changes in epithelial cell-derived Th2 cytokines. *S. aureus* induced reduction of IL-33 expression in nasal epithelium had a crucial impact on the downstream activities of epithelial cell-derived cytokines and suppressed allergic airway inflammation, eosinophil infiltration, and induction of Th2 cytokines such as IL-4, IL-5, and IL-13. This study demonstrates that colonization by *S. epidermidis* and *S. aureus* is highly increased in the nasal mucus of subjects with AR and particularly, inoculation of isolated nasal commensal *S. aureus* suppresses Th2 cytokine-dependent allergic inflammation by regulating epithelial cell-derived cytokines, especially IL-33, in in vitro and in vivo AR models.

There are studies on the effect of the gut microbiome and bacterial administration on Th2 inflammation in AR. Gut microbiome organisms such as *Lactobacilli* and *Bifidobacteria* have recently been found to be effective in murine models of allergic airway inflammation. Animal studies have suggested the effects of gut commensal bacteria on allergen-induced airway responses, and administration of microbiome strains or bacterial components played a protective role in allergic airway inflammation [33, 34].

The nasal mucosa is a key player in immunologic responses to inhaled allergens, and we presumed that local microbiome distribution in the nasal mucus of subjects with AR might influence allergic inflammation. We wonder how nasal *Staphylococcus* species colonization is more abundant in AR nasal mucus and whether this plays a critical role in regulating Th2 immunity and allergic diseases. We surmise that allergic inflammation in nasal mucosa of subjects with AR is thought to provide a better environment for *Staphylococcus* species to colonize and inoculation of *Staphylococcus* species adapted to AR nasal mucus contributed to reduced Th2 inflammatory responses in allergic nasal mucosa. These findings propose that manipulation of the microbiome or inoculation of a dominant nasal microbiome might be a therapeutic strategy for AR.

Among various epithelial cell products, three epithelial cell-derived cytokines, IL-33, IL-25, and TSLP, have critical functions in the pathophysiology of allergic airway diseases. IL-33, predominantly expressed by tissue cells such as epithelial cells, fibroblasts, and endothelial cells, can be induced in allergic inflammatory conditions. By binding suppression of tumorigenicity 2 (ST2), IL-33 activates target cells to produce Th2 cytokines that regulate local and systemic allergic inflammation [6, 33]. Both IL-33 and ST2 are highly expressed in the nasal mucosa of subjects with AR, suggesting that the IL-33/ST2 axis may be a key mechanistic link in the pathogenesis of allergic nasal diseases [6, 33]. We suggest that reduction of IL-33 is a critical option for controlling inflammation in the nasal mucosa of patients with AR, and research to identify an effective modulator of IL-33 may improve the possibility of treating AR. We found that nasal commensal *S. aureus* isolated from subjects with AR preferentially modulated IL-33 expression in both ARNE cells and the nasal mucosa of an OVA-driven AR mice model. The current data indicate that isolation of *S. aureus* from the nasal mucus of subjects with AR and culturing it for intranasal inoculation is a promising therapeutic option for restricting IL-33/ST signaling pathways in AR nasal mucosa. Although *S. aureus* may cause respiratory infections, isolation of *S. aureus* from the adapted environment of AR nasal mucus reduced Th2 inflammation by suppressing IL-33 expression in allergic nasal epithelium.

Our study increases understanding of whether the nasal microbiome of subjects with AR reduces Th2 inflammation to protect AR nasal epithelium from IL-33-mediated inflammation. We showed that abundant *S. aureus* colonization enhances resistance to allergic inflammation in human nasal epithelium by reducing IL-33 expression in nasal mucosa. Thus, intranasal delivery of human nasal commensal *S. aureus* represents a potential therapeutic approach for preventing and treating allergic inflammation via reduction of epithelial cell-derived Th2 cytokines.

## Supplementary information

**Additional file 1.** A video file of the nasal swab for sample collection.

**Additional file 2.** Supplementary material and method.

**Additional file 3.** Supplementary figure.

## Data Availability

The data that support the findings of the current study are available from the corresponding author upon reasonable request.

## Abbreviations

AR: Allergic rhinitis
AR-SA: *Staphylococcus aureus*
AR-SE: *Staphylococcus epidermidis*
NHNE: Normal human nasal epithelium
ARNE: Allergic rhinitis nasal epithelium
TSLP: Thymic stromal lymphopoietin
IL-33: Interleukin-33
Th2: T helper 2
Tregs: Regulatory T cells
