# Supplementary material for: Symbiotic microbiome Staphylococcus aureus from human nasal mucus modulates IL-33-mediated type 2 immune responses in allergic nasal mucosa
Source: BMC Microbiol. 2020 Oct 7;20:301. doi: 10.1186/s12866-020-01974-6 (PMC7542126; doi:10.1186/s12866-020-01974-6)
Supplement: Supplementary file 2 — Additional file 2. Supplementary material and method. [file 12866_2020_1974_MOESM2_ESM.doc]

**Symbiotic microbiome Staphylococcus aureus from human nasal mucus modulates IL-33-mediated type 2 immune responses in allergic nasal mucosa**

Yung Jin Jeon, MD, Chan Hee Gil, Jina Won, Ara Jo, Hyun Jik Kim, MD, PhD

**Materials and methods**

**Participant recruitment and Sample collection**

We recruited 17 patients referred to the Department of Otorhinolaryngology primarily for septal surgery in Seoul National University Hospital (Seoul, Republic of Korea) between October 2017 and September 2018, and in Gyeongsang National University Hospital (Jinju, Republic of Korea) between June 2019 and October 2019. This study was approved and monitored by the Institutional Review Board (IRB) of Seoul National University College of Medicine (No. 1709-049-883) and Gyeongsang National University Hospital (No. 2019-05-004). All subjects who participated in sampling of nasal mucosa provided written informed consent. Septal deviation was diagnosed with intranasal endoscope and paranasal sinus computed tomography (PNS-CT), and the subjects did not show any clinical or imaging findings about sinusitis. To confirm allergic rhinitis (AR), they underwent an allergic skin test (AST) or a multi-allergen simultaneous test (MAST) for the detection of allergens and specific IgEs. Twenty subjects were classified into healthy subjects and fifteen were diagnosed with AR. The mean age of the subjects was 35.2 ± 7.1 years and there was no significant difference between AR and healthy subjects. 1×1 ㎠ sized nasal mucosa was obtained from the middle turbinate of subjects under general anesthesia.

**Cell culture**

Human nasal epithelial cells were cultured as described previously [21]. Briefly, passage-2 NHNE cells (1×10^5^ cells/culture) were seeded in 0.5㎖ of culture medium on Transwell clear culture inserts (24.5㎜, with a 0.45㎜ pore size; Costar Co., Cambridge, MA, USA). Cells were cultured in a 1:1 mixture of basal epithelial growth medium and Dulbecco’s Modified Eagle’s Medium (DMEM) containing previously described supplements. Cultures were grown submerged for the first nine days. The culture medium was changed on day 1, and every other day thereafter. An air–liquid interface (ALI) was created on day 9 by removing the apical medium and feeding the cultures from the basal compartment only. The culture medium was changed daily after the initiation of the ALI. We added antibiotics (such as 1% penicillin and streptomycin) into the media for subculture, and we also added the antifungal agent Fungizone® (1㎖/1000㎖ media; Life technologies, Grand island, NY) after filtering the media. During the last seven days, a basal compartment feeding medium without antibiotics or antifungals was used for the incubation of NHNE cells with *Staphylococcus* species. All experiments described here used cultured nasal epithelial cells taken 14 days after the creation of the ALI.

Isolated *Staphylococcus aureus* strain and *Staphylococcus epidermidis* strain from a patient with allergic rhinitis (AR-SA and AR-SE) were used to induce acute bacterial infection in NHNE and ARNE cells. The AR-SA and AR-SE strains were maintained in a -80°C deep freezer until required, and was plated and grown overnight at 37°C on Luria–Bertani (LB) agar plates (Difco™ LB agar, Miller base; Becton Dickinson and Company, Franklin Lakes, NJ, USA). A single colony of AR-SA and AR-SE was grown in 1㎖ of LB medium (Difco™ LB broth, Miller base; Becton Dickinson and Company, Franklin Lakes, NJ, USA) for 24h, in a 37°C incubation shaker. 10㎖ of fresh LB medium was added to 100㎕ of the AR-SA and AR-SE culture. This mixture was grown for 2h to reach log phase. Passage-2 fully differentiated NHNE cells were either mock-infected (PBS) or inoculated with AR-SA and AR-SE strains to apical side of ALI at a multiplicity of infection (MOI) of 0.25. After inoculation, the cells were incubated at 37°C in 5% CO2. At the designated times post-inoculation, the cell lysate and culture supernatant were collected. The AR-SA and AR-SE cultures were diluted with the non-antibiotic feeding medium to adjust the amount to 300㎕ and inoculated to the apical compartment of each NHNE and ARNE cell culture wells. NHNE and ARNE cells were infected with AR-SA and AR-SE cultures for 0, 2, 8, 24, and 48 hrs.

**Murine inoculation model**

Animal experiments were approved by the Institutional Animal Care and Use Committees of Seoul National University Hospital (No 2016-1470) and the research methods were carried out in accordance with the approved guidelines. Four-week-old female wildtype (WT) BALB/c mice (Orient, Gyeonggi, Republic of Korea) were maintained under specific-pathogen-free conditions, and all mice were housed in a temperature controlled environment with a 12-hour dark/light cycle. For infections, AR-SA and AR-SE (3.2x10^6^ CFU in 30㎕ PBS) were infected into WT mice by intranasal delivery. Mice were euthanized and sacrificed by cervical dislocation and by intramuscular injection of high dose of a mixture of 10mg/kg xylazine (Bayer, Puteaux, France) and 5mg/kg ketamine (Merial, Lyon, France) according to the reviewed protocol. Death was verified when no heartbeat was detected. There were no mice without euthanasia. When mice were euthanized by injection, a cervical dislocation was also performed to ensure that the mice were dead.

After euthanizing the mice, nasal lavage (NAL) fluid was obtained from the nasal cavity by lavaging with 1000μl 0.5mM ethylene diamine tetraacetic acid (EDTA) in phosphate-buffered saline (PBS). The NAL fluid was used for enzyme-linked immunosorbent assay (ELISA) for measuring secreted protein levels. Mouse nasal tissue was also harvested for real-time PCR (RT-PCR),

**Ovalbumin sensitization and nasal challenge**

The mice were divided into four groups. The negative control group (WT-PBS) was sensitized and challenged with phosphate-buffered saline (PBS), the positive control group (WT-OVA) was sensitized and challenged with ovalbumin (OVA), the SA-PBS group consisted of AR-SA infected mice sensitized and challenged with PBS, and the SA-OVA group consisted of AR-SA infected mice sensitized and challenged with OVA. The schedule for allergen sensitization and intranasal challenge is summarized in figure 1A. Briefly, the WT-OVA and SA-OVA groups were sensitized by intraperitoneal injection of 25㎍ OVA mixed with 2㎎ alum on days 0, 7, and 14 and then challenged by intranasal treatment of 100㎍ OVA for 7 consecutive days, from days 22 to day 28. The WT-PBS and SA-PBS groups were injected intraperitoneally and challenged intranasally with PBS following the same schedule.

**Serum levels of total and OVA-specific IgE**

Mouse serum samples were stored at -70℃ before using for measurements of total IgE and OVA specific IgE, as described previously [22]. Briefly, total serum IgE was measured by a standard enzyme-linked immunosorbent assay (ELISA) using an anti-mouse IgE capture monoclonal antibody (BD Pharmingen, San Diego, CA, USA) and horseradish peroxidase (HRP)-conjugated anti-mouse IgE (Southern Biotechnology, Birmingham, AL, USA). To detect OVA-specific IgE, 96-well immune plates were coated with 100㎍/㎖ of OVA in carbonatebicarbonate buffer. After the serum samples had been incubated for 2h, biotin-conjugated rat anti-mouse IgE monoclonal antibody (BD Pharmingen) and streptavidin-HRP (BD Pharmingen) were used to detect OVA-specific IgE levels.

**Real-time PCR and RNA preparation**

Total RNA was isolated using TRIzol (Life technology, Seoul, Republic of Korea) and cDNA was synthesized from 3μg of RNA with random hexamer primers and Moloney murine leukemia virus reverse transcriptase (Perkin Elmer Life Sciences, Waltham, MA, USA and Roche Applied Science, Indianapolis, IN, USA). Amplification was performed using the TaqMan Universal PCR Master Mix (PE Biosystems, Foster City, CA, USA) according to the manufacturer’s protocol. Briefly, amplification reactions had a total volume of 12μl and contained 2μl of cDNA (reverse transcription mixture), oligonucleotide primers (final concentration of 800nM), and TaqMan hybridization probe (200nM). RT-PCR probes were labeled at the 5’ end with carboxyfluorescein (FAM) and at the 3’ end with the quencher carboxytetramethylrhodamine (TAMRA). To quantify the cellular viral level and host gene expression, cellular RNA was used to generate cDNA.

The AR-SA and AR-SE levels were monitored using a RT-PCR for the factor essential for the expression of methicillin resistance (*femA*) genes specific for *S. aureus* and *S. epidermidis*. The forward and reverse primers and probes used for real-time are listed in supplementary table 1. Primers for human IL-33 and TSLP, mouse IL-4, IL-5, IL-13 and IL-33 were purchased from Applied Biosystems (Foster City, CA, USA). RT-PCR was performed using the PE Biosystems ABI PRISM® 7700 Sequence Detection System. Thermocyling parameters were as follows: 50°C for 2 min, 95°C for 10 min, and then 40 cycles of 95°C for 15 s and 60°C for 1 min. All RT-PCR assays were quantitative and utilized plasmids containing the target gene sequences as standards. All reactions were performed in triplicate, and all RT-PCR data were normalized to the level of the housekeeping gene glyceraldehyde phosphate dehydrogenase (GAPDH, 1×10^6^ copies) to correct for variations between samples.

Table 1: Real-time PCR primers and TaqMan probes for *femA*s specific for *S. aureus* (*femA-SA*) and *S. epidermidis* (*femA-SE*).

| Target genes | Sequence |
| --- | --- |
| *femA-SA* | 5′-TGCCTTTACAGATAGCATGCCA-3′ |
|  | 5′-AGTAAGTAAGCAAGCTGCAATGACC-3′ |
|  | 5′-JOE-TCATTTCACGCAAACTGTTGGCCACTATG-BHQ1-3′ |
| *femA-SE* | 5′-CAACTCGATGCAAATCAGCAA-3′ |
|  | 5′-GAACCGCATAGCTCCCTGC-3′ |
|  | 5′-JOE-TACTACGCTGGTGGAACTTCAAATCGTTATCG-BHQ1-3′ |

**Protein isolation and Western blot**

Total protein lysates were harvested in RIPA lysis buffer (Thermo Fisher Scientific, Wilmington, DE, USA). The cell lysates (25μg per lane, as measured by a BCA protein assay purchased from Thermo Fisher Scientific) were electrophoresed in 10% SDS gels and transferred to polyvinylidene difluoride membranes in Tris-buffered saline (TBS; 50mM Tris-Cl, pH 7.5, and 150 mM NaCl) for one hour at room temperature. Each membrane was incubated overnight with primary antibody in Tween-Tris-buffered saline (TTBS; 0.5% Tween-20 in TBS) at 4°C. After washing with TTBS, each blot was incubated for one hour at room temperature with secondary anti-rabbit or anti-mouse antibody (Cell Signaling, Beverly, MA, USA) in TTBS. Expression was detected using an enhanced chemiluminescence system (Amersham, Little Chalfont, UK).
