# Supplementary material for: Symbiotic microbiome Staphylococcus aureus from human nasal mucus modulates IL-33-mediated type 2 immune responses in allergic nasal mucosa
Source: BMC Microbiol. 2020 Oct 7;20:301. doi: 10.1186/s12866-020-01974-6 (PMC7542126; doi:10.1186/s12866-020-01974-6)
Supplement: Supplementary file 3 — Additional file 3. Supplementary figure. [file 12866_2020_1974_MOESM3_ESM.doc]

**Symbiotic microbiome Staphylococcus aureus from human nasal mucus modulates IL-33-mediated type 2 immune responses in allergic nasal mucosa**

Yung Jin Jeon, MD, Chan Hee Gil, Jina Won, Ara Jo, Hyun Jik Kim, MD, PhD

**Fig. 3f. *Staphylococcus* species suppress expression of IL-33 in ARNE cells**

**IL-33 protein level of NHNE (upper panel) and ARNE cells (lower panel) inoculated with AR-SA were analyzed by western blot**

| **NHNE** | *AR-SA* (MOI 0.25) | | | | | | | | | | | | |
| --- | --- | --- | --- | --- | --- | --- | --- | --- | --- | --- | --- | --- | --- |
|  |  | 0 | 2 | 8 | 24 | 48 |  | 0 | 2 | 8 | 24 | 48 |  |


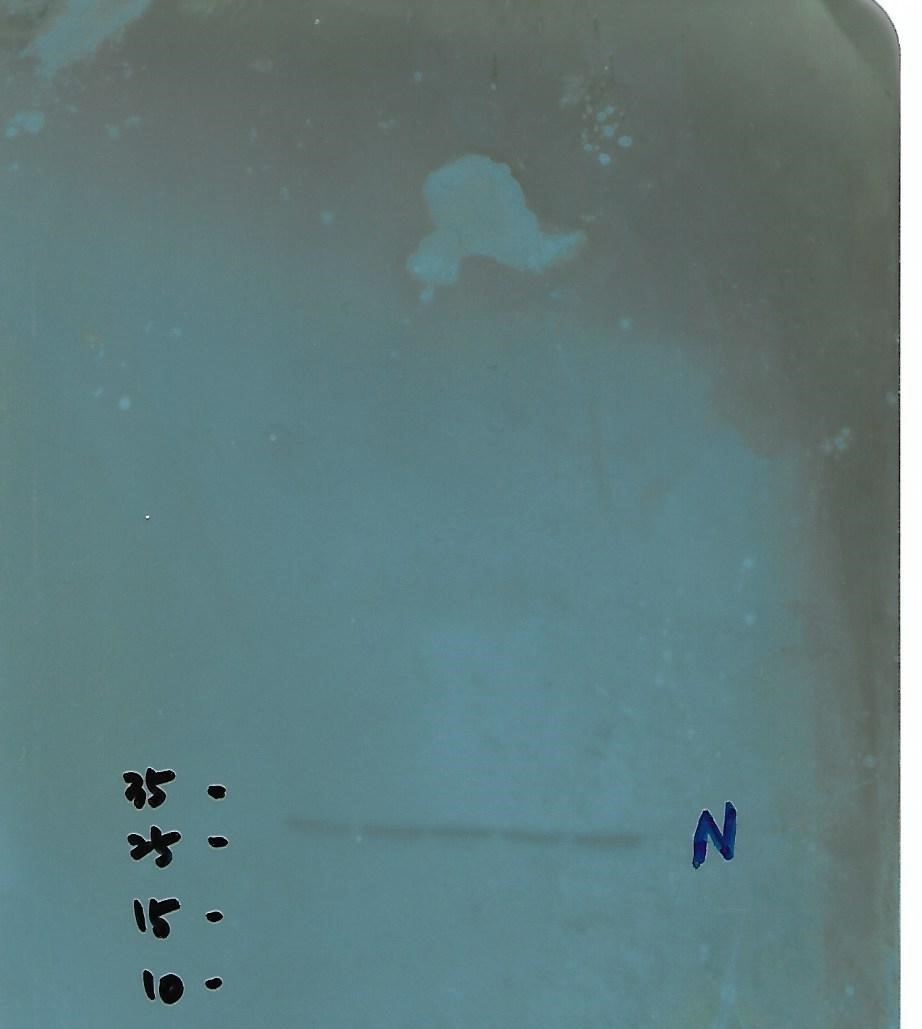

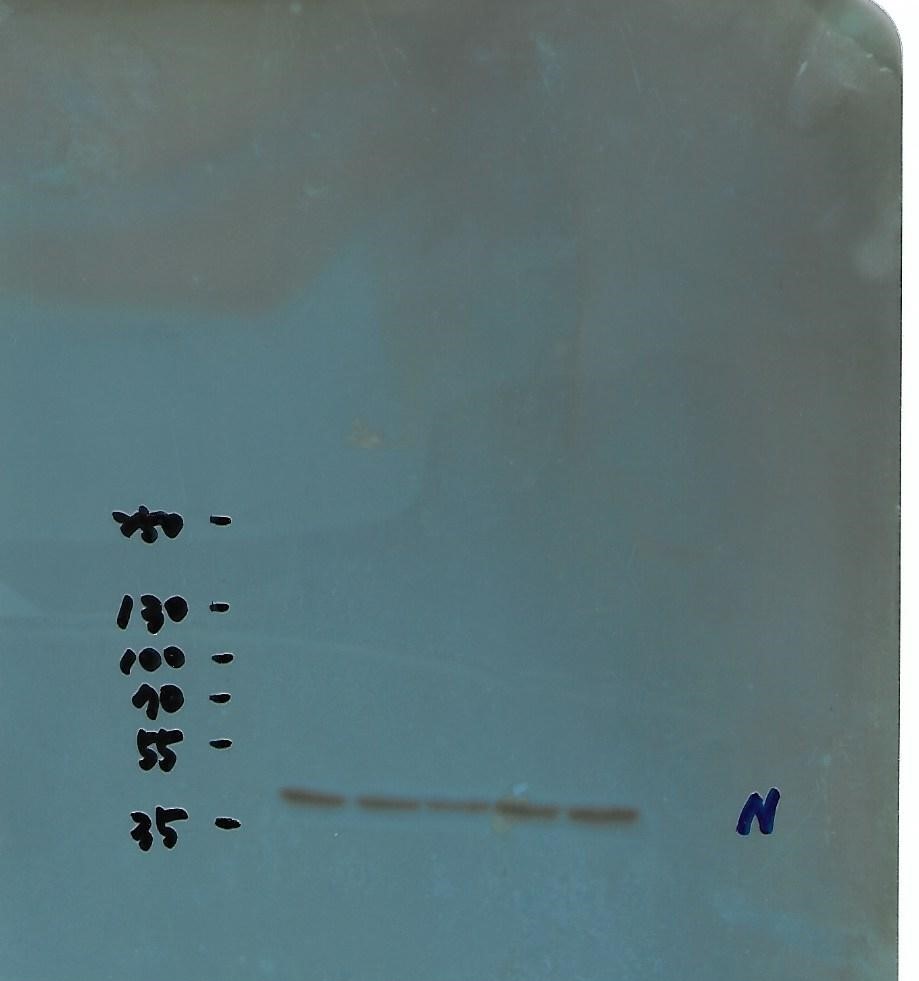


| IL-33 | β-actin |
| --- | --- |

| **ARNE** | *AR-SA* (MOI 0.25) | | | | | | | | | | | | |
| --- | --- | --- | --- | --- | --- | --- | --- | --- | --- | --- | --- | --- | --- |
|  |  | 0 | 2 | 8 | 24 | 48 |  | 0 | 2 | 8 | 24 | 48 |  |


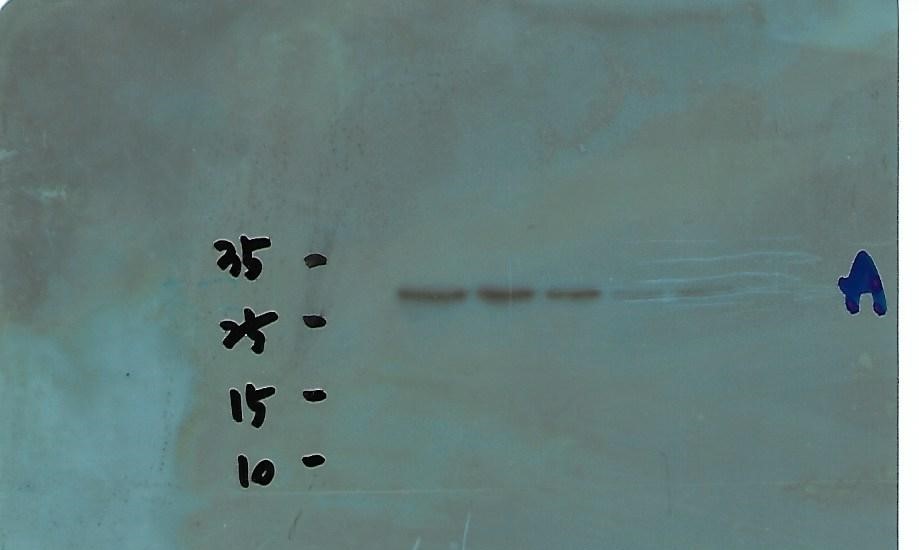

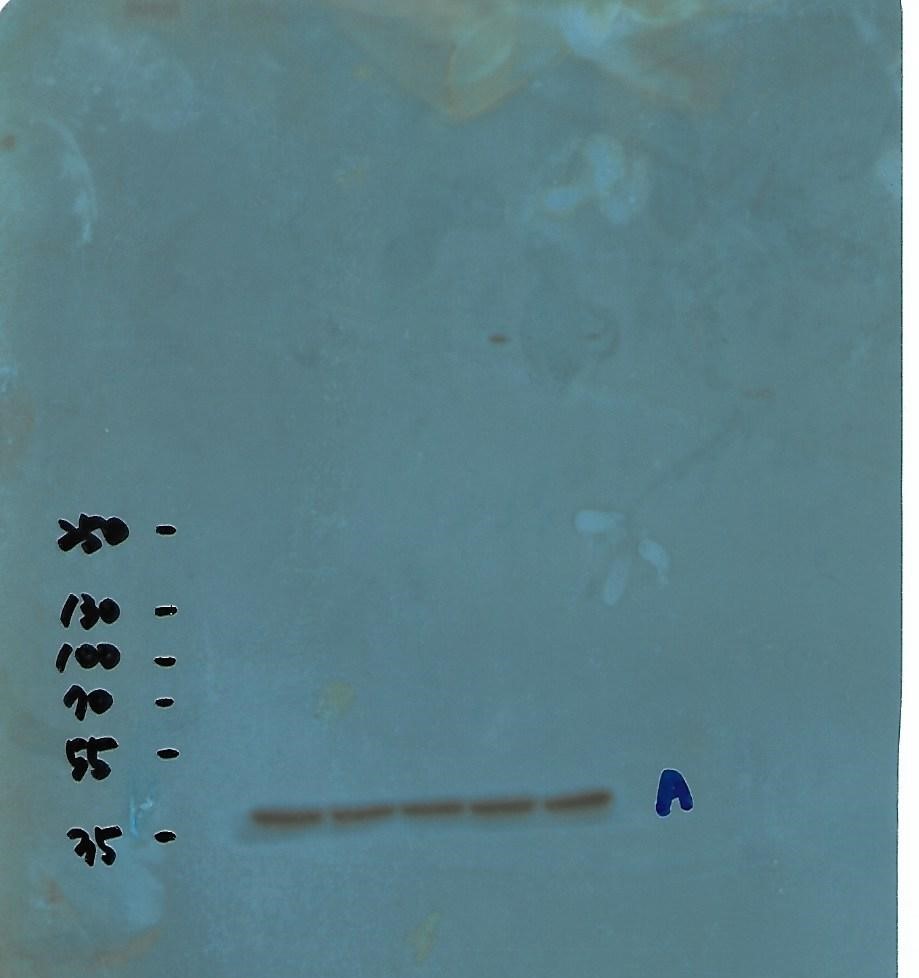


| IL-33 | β-actin |
| --- | --- |
